# Assessment of bacterial profile of drinking water from different sources in Nekemte and their antibiotic susceptibility pattern, Western Ethiopia

**DOI:** 10.1038/s41598-026-49165-z

**Published:** 2026-04-21

**Authors:** Desalegn Amenu, Waktole Gobena, Temesgen Tafesse

**Affiliations:** 1 Food Safety and Anthropology Division, Food Microbiology, Ethiopian Public Health Institute, Addia Ababa, Ethiopia; 2 Armauer Hansen Research Institute, Addis Ababa, Ethiopia

**Keywords:** Bacterial profile, Drinking water, Antibiotic susceptibility pattern, Western Ethiopia, Environmental sciences, Microbiology, Water resources

## Abstract

Safe drinking water is essential for public health, yet microbial contamination remains a challenge in developing countries. This study evaluated the physicochemical and bacteriological quality of drinking water from 30 sites in Nekemte town, Western Ethiopia, including protected/unprotected springs, tap water, and bottled water. A total of 90 samples (triplicates per site) were followed across different seasons. The APHA (2017) and WHO (2021) drinking water standards were compared in the analysis of physicochemical parameters, including pH, temperature, turbidity, total dissolved solids (TDS), total suspended solids (TSS), and electrical conductivity (EC). The pour plate was used for bacteriological analysis. Total coliforms, fecal coliforms, Enterobacteriaceae, and heterotrophic plate counts were counted. Bacterial isolates were typed to the genus level using standard biochemical tests. The antibiotic sensitivity test was conducted by Kirby–Bauer disc diffusion method on Mueller–Hinton agar according to guidelines of CLSI (2012) employing antibiotic discs (10–30 µg) and Escherichia coli ATCC 25,922 as the control. Physicochemical parameters of all samples fell within WHO acceptable limits (pH: 5.13–6.83; TDS: 28–120.5 mg/L). Yet, bacteriological testing showed contaminant levels above safe levels in both spring and tap water samples. Mean total coliform counts varied between 1.04 ± 0.12 to 3.58 ± 0.21 CFU/100mL (ANOVA, *p* < 0.05). The bacterial isolates totaled 57, with the majority being Staphylococcus (26.3%), Pseudomonas (19.3%), and Salmonella (15.8%). Interestingly, 41.7% of the isolates were antibiotic resistant to E. coli. In conclusion, the study confirms that most of the water sources in Nekemte Town are not microbiologically safe for consumption. The study portrays the urgent necessity for improved water treatment, improved community hygiene, and frequent microbial pollution monitoring and antibiotic resistance for public protection.

## Introduction

Safe water for drinking and proper sanitation is key to human health and sustainable development. Waterborne illness across the globe continues to be a significant cause of morbidity and mortality, particularly in low- and middle-income countries, and children younger than five years are most vulnerable. Diarrheal diseases alone cause a high rate of morbidity and mortality from unsafe water and unsanitary sanitation^1^. In Ethiopia, the disease burden of diarrhoeal disease among children below five years of age remains high, and evidence reveals poor access to improved water sources and sanitation facilities as one of the key risk factors for the disease^2,3^. This highlights the long-standing public health challenge of providing safe drinking water in Ethiopian communities and the need for localized water-quality surveys^5^.

Sources of drinking water are polluted through a plethora of anthropogenic and environmental pathways, including municipal effluents, industrial effluent, agricultural runoff, and cross-contamination within piped water supply systems^5,6^. Poorly protected spring water, private tap system leaks, and poor water storage also heighten the risks for microbial contamination^4^. In Ethiopia, studies have revealed that a vast majority of the population still relies on unimproved water sources with widespread variation in water quality in both urban and rural areas^3^. These findings stress the need for intensive water-quality monitoring, particularly in urban towns such as Nekemte, where several sources, including bottled water, private taps, and communal springs, are under wide use.

Traditional water-quality analysis has relied mainly on indicator organisms, for example, total coliforms and faecal coliforms, and broad physicochemical parameters like pH, turbidity, total dissolved solids, and electrical conductivity^1,5^. While useful, this approach may not capture the presence of pathogenic bacteria, protozoa, or viruses and provides no data on antibiotic-resistance profiles of cultivable microorganisms. Recent studies have stressed the importance of including pathogenic bacteria identification and antibiotic susceptibility testing within water-quality surveys to reveal hidden public health risks^4^. This broad surveillance allows for more specific characterization of microbial hazards and targets interventions to protect public health.

Most previous studies in Nekemte Town have focused on either the physicochemical or microbiological quality of a single water source, such as municipal taps or springs^7,8^. Comparative assessments across multiple sources—including bottled water, private taps, and public springs are limited. Moreover, data on antibiotic resistance patterns of bacterial isolates from these water sources are lacking, despite the role of drinking water as a potential reservoir for multidrug-resistant bacteria and its contribution to the wider spread of antibiotic resistance^4,5^.

This study addresses these gaps by providing a comprehensive assessment of water quality across multiple source types and seasons, integrating both microbiological and physicochemical analyses. By evaluating indicators such as total coliforms, fecal coliforms, Enterobacteriaceae, antimicrobial resistance patterns, and key physicochemical parameters, the study not only characterizes local water quality but also generates insights relevant to other urban and peri-urban areas in Ethiopia and similar low-resource settings. Comprehensive, multi-source, seasonally stratified studies of this kind are still relatively scarce, and our work extends existing evidence by offering a framework that can inform broader water safety management and public health strategies beyond Nekemte^9^.

The study’s implications include providing a general conclusion regarding the quality of water in Nekemte Town. By analyzing physicochemical parameters, bacterial contamination, and antibiotic-resistance profiles across different water sources, this research offers an integrated view of potential health risks from local water consumption. The findings can guide source-specific interventions, strengthen regulatory control over bottled water, and support public health education campaigns^1,2^. Additionally, the results contribute to the growing body of scientific evidence on drinking water quality in urban Ethiopian contexts, supporting evidence-based policy-making at local and regional levels^2^. Therefore, this study aimed to determine the bacteriological and physicochemical quality of different water sources in Nekemte, and assess the antibiotic susceptibility of major bacterial isolates. Finally, the present study will give a general evaluation of the physicochemical and microbial quality of bottled water, private tap water, and communal spring water in Nekemte Town, Ethiopia, along with the examination of the antibiotic-resistance patterns of the bacterial isolates. By filling existing knowledge gaps with the literature, this research will provide operationally useful source-specific risk information, directly inform local public health response, and national and regional water-quality control and antibiotic resistance prevention policy^1–5^.

## Materials and methods

### Description of the study area

**Fig. 1:**
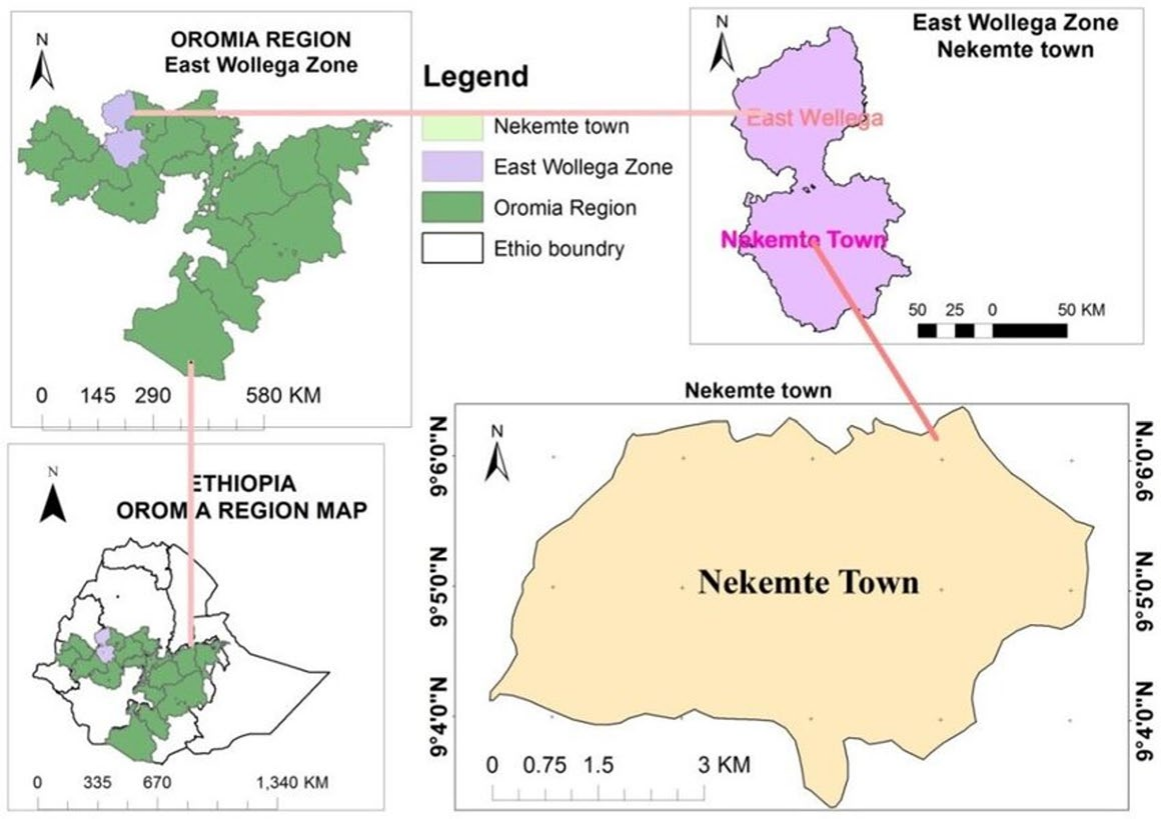
Study area (East Wollega Zone, Nekemte town, Desalegn et al., 2024).

It was conducted in Nekemte town, capital of East Wollega Zone in Western Ethiopia, with 9.083°N latitude and 36.550°E longitude and 2,088 m above sea level. The town has an estimated population of 110,688 (2012 census) and a Weyena Dega type temperate climate with relatively low temperature. Most of the rainfall occurs during June, July, and August, and fairly less rainfall is observed in April, May, and September. High rainfall promotes agriculture with irrigated farming relying on surrounding rivers. The water bodies are often exposed to sewage discharge, domestic sewage, and agricultural runoff, which can introduce microbial and chemical pollutants that have the potential to be harmful to public health. These anthropogenic and environmental factors make Nekemte a suitable site for water quality determination both in microbiological safety and physicochemical parameters (Fig. 1).

### Sample size, sampling points, and sampling procedures

A total of 90 water samples were collected from the three main drinking water sources in Nekemte town: bottled water (30 samples), private taps (30 samples), and springs (protected and unprotected, 30 samples). Triplicate samples were collected at each site to ensure reproducibility and statistical reliability. Sampling sites were stratified by water source type and randomly selected across different neighborhoods to provide a representative overview of water quality in Nekemte, rather than focusing solely on areas near potential contamination. Sites were selected to include both locations near potential contamination sources—such as agricultural runoff or sewage discharge and relatively protected areas, ensuring spatial representativeness. Geographic coordinates of each site were recorded using GPS, and a map of all sampling locations is provided in Fig. 1 to illustrate the spatial distribution.

To account for temporal variation, samples were collected during both wet and dry seasons. Water was collected in sterilized 500 mL plastic bottles, labeled, and transported on ice (4 °C) to the Wollega University Laboratory. Aseptic techniques were strictly applied during collection, handling, and transport to prevent contamination. Quality assurance procedures included the use of field blanks and duplicate samples to monitor potential contamination during sampling and transportation. This study used a cross-sectional study design to assess the bacterial profile of drinking water from different sources in Nekemte town, Western Ethiopia, and to determine the antibiotic susceptibility patterns of the isolated bacterial pathogens.

Water samples were collected from 30 sites across Nekemte town, including 10 bottled water brands, 10 private taps from different neighborhoods, and 10 springs (protected and unprotected). Three replicate samples were collected at each site, totaling 90 samples. The sites were selected to represent the main water sources used by the community, covering both areas near potential contamination (e.g., sewage discharge, agricultural runoff) and more protected locations. Geographic coordinates of all sites were recorded using GPS, and a map of all sampling locations is provided in Fig. 1. Tables 1, 2 and 3 summarize the microbiological and physicochemical results for each site, with sample numbers indicated.

### Microbial analysis

The microbial quality of water samples was assessed using the pour plate technique. Indicator microorganisms such as total heterotrophic bacteria, Enterobacteriaceae, total coliforms, and fecal coliforms were enumerated after incubation, and bacterial counts were recorded as colony-forming units per milliliter (CFU/100mL). The collected water samples were serially diluted using sterile peptone water and analyzed using the pour plate technique. Appropriate dilutions of the water samples were inoculated into sterile Petri dishes and plated on selective and differential media, including nutrient agar, MacConkey agar, and m-Endo agar, for the enumeration and isolation of bacterial groups. The plates were incubated at appropriate temperatures depending on the target microorganisms: 35 °C for 24–48 h for total heterotrophic bacteria and 37 °C for 24 h for coliform bacteria. After incubation, visible colonies were counted and the results were expressed in logarithmic form as colony-forming units per milliliter (log CFU/100 mL).

Indicator microorganisms, including total heterotrophic bacteria, Enterobacteriaceae, total coliforms, and fecal coliforms, were used as indicators of microbial and fecal contamination of the water sources. Quality control procedures were maintained throughout the analysis. Standard reference strains such as Escherichia coli ATCC 25922 and Enterococcus faecalis ATCC 29212 were used to verify the performance of culture media and biochemical tests. Reagent blanks were also included to ensure sterility and validate the culture procedures^10^.

Bacterial isolates were characterized by colony morphology, Gram staining, and standard biochemical tests (catalase, oxidase, coagulase, urease, citrate, indole, and MR-VP) to identify them to the genus level. Identification was based on biochemical profiles compared with reference charts from *Bergey’s Manual of Systematic Bacteriology*. No molecular methods or species-level identification were performed.

### Pathogen isolation and identification

To determine the presence of pathogens in water, bacterial isolates were obtained through selective enrichment and cultivation on appropriate agar media. Identification of these isolates to genus and species levels was conducted using a combination of approaches, including morphological characteristics (colony color, size, shape, and Gram staining), biochemical tests (catalase, oxidase, sugar fermentation, and IMViC tests), and physiological traits (sodium chloride tolerance and growth at 4–42 °C). This comprehensive and systematic approach ensured reliable and robust identification of potentially pathogenic bacteria associated with drinking water^11^.

### Antibiotics susceptibility testing

Kirby-Bauer disk diffusion test was utilized to determine bacterial isolate susceptibility, as per CLSI (2012) recommendations. Enterobacteriaceae and Streptococci isolates were tested against eight drugs: chloramphenicol (30 µg), ciprofloxacin (5 µg), erythromycin (15 µg), gentamicin (10 µg), kanamycin (30 µg), penicillin (10 units), streptomycin (10 µg), and tetracycline (30 µg). Repeatability was guaranteed by the inclusion of quality control strains like Escherichia coli ATCC 25,922 and Streptococcus pneumoniae ATCC 49619. Inhibition zones were measured in millimeters, and results were evaluated according to CLSI breakpoints, which provided information about possible antibiotic resistance of waterborne bacteria^12^.

### Physicochemical analysis

**Fig. 2:**
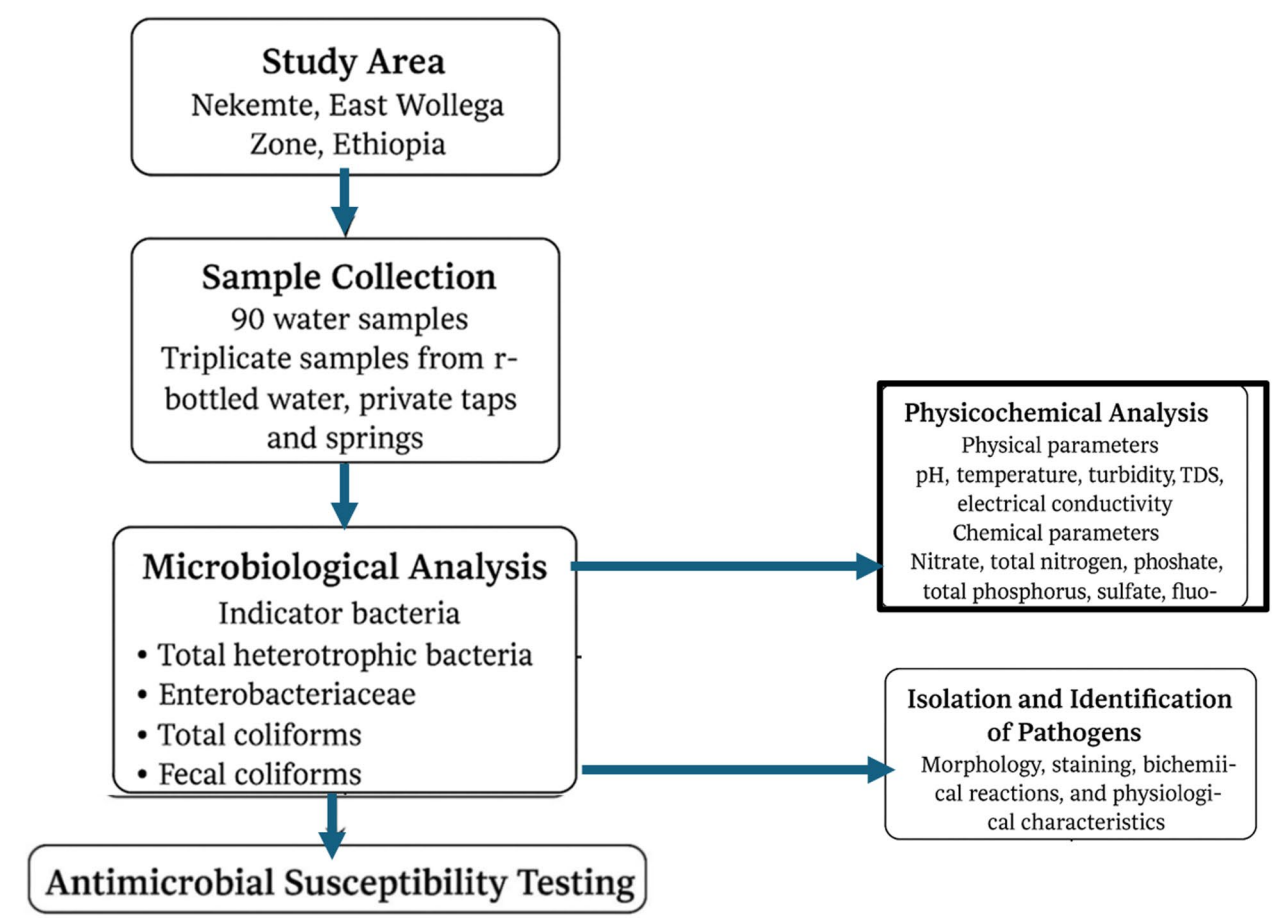
Study design and procedural activities flow diagram (flow chart).

Physicochemical characteristics of water samples were determined by standard procedure as suggested by APHA and HACH. Physical parameters included pH (determined by a calibrated pH meter), temperature in °C (with thermometer), total dissolved solids (TDS, mg/L by a TDS meter), turbidity (NTU by the utilization of a turbidity meter), and electrical conductivity (EC, µS/cm by an EC meter). Chemical parameters involved nitrate (NO₃⁻, mg/L by HACH DR spectrophotometer), total nitrogen (TN, mg/L by Kjeldahl method), phosphate (PO₄³⁻, mg/L by ascorbic acid method), total phosphorus (TP, mg/L by colorimetric method), sulfate (SO₄²⁻, mg/L by turbidimetric method), and fluoride (F⁻, mg/L by an ion-selective electrode). Each analysis was executed by properly calibrated equipment and confirmed laboratory practices for the assurance of accuracy and reproducibility^13^. Laboratory protocols are listed in below flow diagram (Fig. 2).

### Data analysis

Data were analyzed using descriptive statistics (mean ± standard deviation). Normality of data was tested prior to parametric analyses. One-way ANOVA was performed to compare microbial and physicochemical parameters across water sources, followed by Tukey’s post-hoc test for pairwise comparisons. Pearson correlation analysis was conducted to explore relationships between physicochemical parameters and microbial loads. Significance was set at *p* < 0.05. All analyses were performed using SPSS version 25.

## Results and discussion

### Bacteriological quality analysis of water sources

Standard microbial indicators like *Total Coliforms*, *Fecal Coliforms*,* Enterobacteriaceae*, and *Heterotrophic Plate Count* were used to monitor the bacteriological quality of different water sources in Nekemte town, Ethiopia. The aim was to identify the major sources of bacterial contamination and assess possible public health risks. The analysis showed that nearly all water samples were contaminated with **TC**,** FC**,** EBC**, and **HPC**, which serves as an indication of widespread pollution. Generally, natural springs, even those at Cheleleki, Darge, and Jato, have shown higher bacterial loads compared to tap and bottled water supply sources, though fecal indicators were even present in protected springs. On the contrary, bottled water had no detectable bacteria, while tap water in general had safer microbial standards (Table 1).

**Table 1:** Bacterial load of water samples collected Bottled water, Tap water, unprotected and springs in Nekemte town, 2017(CFU/100 ml).

Site	Water sources	Heterotrophic bacteria (log CFU/ 100mL)	Enterobacteriaceae (log CFU/100mL)	Total coliform (log CFU/100mL)	Fecal coliform (log CFU/100mL)
Cheleliki	Tap water	4.01 ± 0.42^a^	1.68 ± 0.25^def^	2.27 ± 0.01^cd^	0.00 ± 0.00
	Unprotected spring	4.13 ± 0.54^a^	3.01 ± 0.42^bc^	3.58 ± 0.20^a^	2.49 ± 0.01^ab^
	Protected spring	4.39 ± 0.72^a^	2.91 ± 0.36^cd^	2.73 ± 0.02^cd^	2.83 ± 0.01^a^
Darge	Tap water	3.57 ± 0.41^b^	2.53 ± 0.58^cd^	2.05 ± 0.05^cde^	2.55 ± 0.02^ab^
	Unprotected spring	4.27 ± 0.75^a^	3.21 ± 0.25^a^	3.18 ± 0.02^ab^	2.89 ± 0.02^a^
	Protected spring	3.54 ± 0.82^b^	1.97 ± 0.47^de^	1.04 ± 0.20^e^	1.34 ± 0.01^de^
Jato	Tap water	3.95 ± 0.94^ab^	2.85 ± 0.36^cd^	2.76 ± 0.20^cd^	2.59 ± 0.36^ab^
	Unprotected spring	2.62 ± 0.63^cd^	1.83 ± 0.48^de^	1.11 ± 0.14^e^	2.33 ± 0.02^b^
	Protected spring	4.45 ± 0.69^a^	4.19 ± 0.50^a^	1.09 ± 0.15^e^	2.40 ± 0.01^b^
Bottled water	Yes, water brand	0.00 ± 0.00^g^	0.00 ± 0.00^f^	0.00 ± 0.00^f^	0.00 ± 0.00^e^
	Blue water brand	0.00 ± 0.00^g^	0.00 ± 0.00^f^	0.00 ± 0.00^f^	0.00 ± 0.00^e^
	Aqua water brand	0.00 ± 0.00^g^	0.00 ± 0.00^f^	0.00 ± 0.00^f^	0.00 ± 0.00^e^

Note: Means with the same superscript letters within a column are not significantly different (*p* > 0.05), based on one-way ANOVA followed by Tukey’s HSD post-hoc test.

**Fig. 3:**
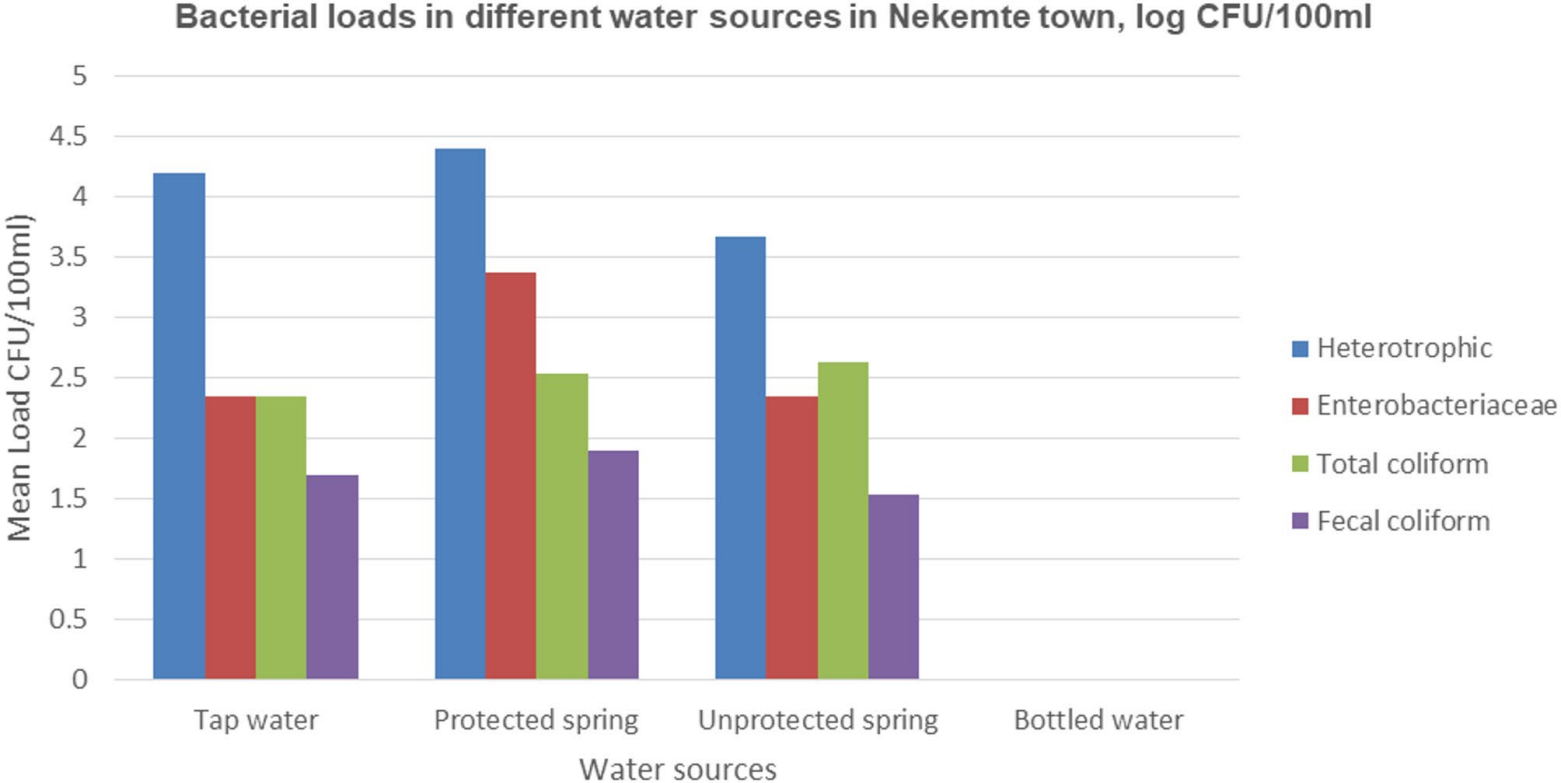
Bacterial load comparison among different water sources.

Out of all studied samples, the counts of total coliforms and fecal coliforms were highest from Cheleleki protected spring and Hawato tap water, respectively. In addition, in the studied samples, the fecal coliform count was lowest in Hawato tap water, while the maximum load was measured in Jato protected spring. The count of HPCs was similarly highest in Hawato protected spring with 282.33 CFU/ml and lowest in Jato protected spring, with 34 CFU/ml. Similarly, *Enterococcus* load was highest in the Hawato protected spring. Although some of these springs have been declared as protected, in general high loads of fecal indicator bacteria suggest that these water sources are still susceptible to contamination. This contamination could be related both to organic deposits from human and animal sewage and to high levels of suspended solids, as shown in Fig. 3.

All the water samples had total heterotrophic counts above the WHO recommended limit of 1.0 × 10² CFU/100 ml. Total coliform levels were above the recommended zero MPN per 100 ml guideline for WHO drinking water. Fecal coliform counts were also above the maximum allowable limit of 14 CFU/100 mL for shellfish-harvesting waters, according to USEPA standards^19,20^. The implication of these findings is that the consumptive use or utilization of these untreated spring and protected spring water sources in Nekemte town poses a serious public health risk. The main bacterial contaminants were members of the *Enterobacteriaceae* family, which comprise Gram-negative, non-spore-forming bacilli in which several pathogenic species are known to occur^14^.

These results are supported by other studies conducted in various parts of Ethiopia, where even the supposedly protected natural water sources present high microbial contamination owing to anthropogenic activities [21.14]. Relatively higher bacterial loads were determined for spring waters compared to tap and bottled waters, hence infrastructures and treatment processes are found to significantly reduce microbial risks. In addition, the high prevalence of coliforms calls for routine monitoring, efficient improvement of water treatment processes, and/or protection strategies at the community level to minimize contamination in ways that ensure public health.

**Fig. 4:**
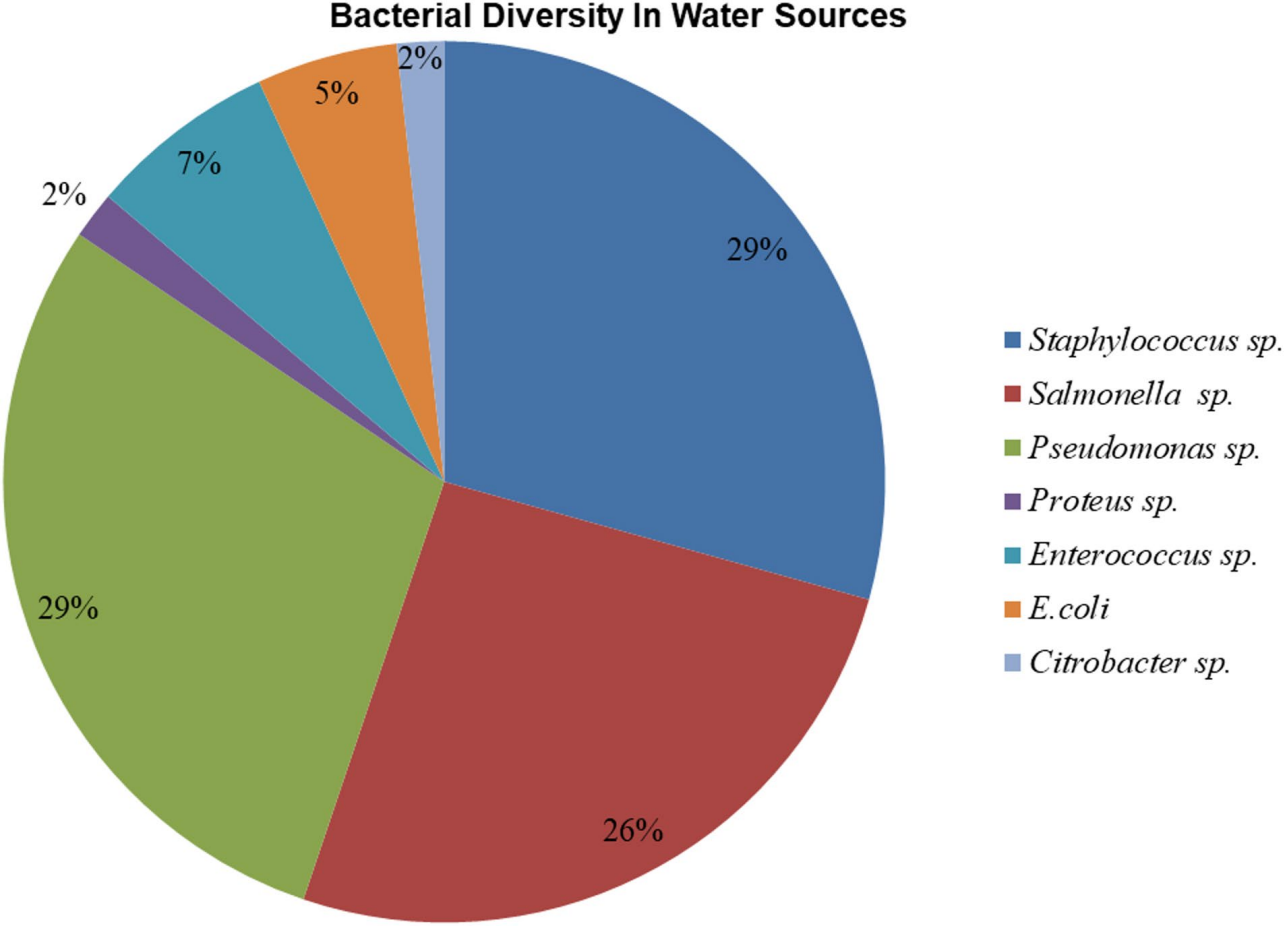
Bacteria diversity in water sources.

While it’s recognized that coliform bacteria are naturally occurring and not exclusively indicative of fecal pollution, the presence of specific species like *E. coli*, *Citobacter*, and *Klebsiella* known indicators of fecal contamination is particularly concerning. Additionally, Gram-positive cocci like *Staphylococcus aureus*, which are fecal streptococci, and enterococci strains like *S. feacalis*, commonly found in humans and animals, were also isolated during the study, further raising concerns about waterborne pathogens (Fig. 4). Overall, the presence of these various bacteria in water sources highlights the potential risks associated with fecal contamination and the importance of monitoring and addressing water quality issues. It is crucial to implement effective water treatment and sanitation measures to prevent the spread of waterborne diseases and protect public health.

This study also determined antibiotic resistances among the bacterial isolates, including *Salmonella*,* Citrobacter sp.*,* Pseudomonas sp*., and *Staphylococcus* sp., which corroborates several works reported in Ethiopia and elsewhere. Among these, Ciprofloxacin was highly effective against *Staphylococcus* sp. and *Enterococcus* sp., while Salmonella typhi showed resistance to chloramphenicol, ampicillin, and trimethoprim, reflecting resistant outbreaks from across the world. Resistance levels of Salmonella in this study correspond to such patterns, ranging between 20 and 47%. (Table 2). Similarly, antibiotics resistance patterns of bacterial isolates from water sources are shown in below Table 3.

**Table 2:** Antibiotics resistance patterns of Gram bacterial isolates from water sources in Nekemte town, 2017.

Isolates	Oxa.	Cip.	C	Aml.	Amp.	Ery.	Gen.
Citrobacter sp.	R	I	R	R	R	R	I
E. coli (3)	R	I	I	R	R	R	I
Enterococcus sp. (4)	R	S	I	R	R	R	I
Proteus sp.	R	I	I	R	R	R	R
Pseudomonas sp. (17)	R	I	R	R	R	R	I
Salmonella sp. (14)	R	R	I	R	R	R	R
Staphylococcus sp. (18)	R	S	I	R	R	R	I

Oxa. = Oxacillin; Cip. = Ciprofloxacin; C = Chloramphenicol; Aml. = Amoxicillin; Amp. = Ampicillin; Ery. = Erythromycin; Gen. = Gentamicin; R = Resistant; S = Sensitive; I = Intermediate; Total Isolates = number of isolates tested per bacterial species.

**Table 3:** Antibiotics resistance patterns of Gram bacterial isolates from water sources in Nekemte town, 2017.

Antibiotic disks	Total isolates tested	Sensitive (S)	Resistant (*R*)	Intermediate (I)
Oxacillin (Oxa.)	58	0	45	13
Ciprofloxacin (Cip.)	58	4	43	11
Chloramphenicol (C)	58	10	43	5
Amoxicillin (Aml.)	58	0	45	13
Ampicillin (Amp.)	58	0	45	13
Erythromycin (Ery.)	58	0	45	13
Gentamicin (Gen.)	58	4	45	9

These findings point out the risk to public health posed by antibiotics resistance microorganisms present in environmental sources. Similarly, such wastewater ponds in Ethiopia act as a reservoir for ARB and ARGs, hence contribute to their dissemination into natural water bodies. The conventional WWTPs often cannot completely remove ARBs and ARGs. Therefore, residual resistant bacteria and antibiotics from these treatment plants reach the environment, contributing to growing resistance across microbial populations^21,22^. Apart from wastewater, agricultural activities, especially raising livestock, continue to be among the major drivers of environmental AMR. In addition to the huge usage in both therapeutic and non-therapeutic ways, manure is concentrated with ARGs three to five orders higher compared to urban and hospital wastewater^23–25^. This dissemination gets enhanced through irrigation with untreated water, night soil used for fertilizer, and urban sewage systems that facilitate exchange of resistance genes among microbial species^26,27^. The presence of ARB in environmental reservoirs presents direct risks to human health through difficult-to-treat infections arising from exposure by water, food, or contaminated direct contact^28,29^.

Regarding to Nekemte water quality, the present study established that bottled water was free from microbial contamination as a result of efficient treatment and source protection, besides regulatory oversight. Tap and surface water sources showed significant bacterial contamination, indicating poor sanitation and environmental exposure^30,31^. Antibiotic susceptibility testing among the isolates from these sources revealed high resistance to commonly used antibiotics such as Oxacillin, Amoxicillin, Ampicillin, and Erythromycin, whereas Ciprofloxacin, Chloramphenicol, and Gentamicin showed partial effectiveness. This presence of multi-drug-resistant bacteria points to the possibility of waterborne dissemination of resistant pathogens and thus suggests a reasonable need for the use of antibiotics and stewardship of antimicrobials^32,33^.

The results thus indicate that both environmental and surface waters in Nekemte town play the role of reservoirs and transmission pathways of antibiotic-resistant bacteria, reflecting the global evidence for AMR spread through human, agriculture, and wastewater sources. Contamination of natural and tap water sources is directly related to the multi-drug resistance patterns observed here, reflecting how environmental exposure contributes to resistance in clinically relevant pathogens. The safety of bottled water further demonstrates how proper treatment and source protection prevent microbial contamination. These findings taken together thus indicate urgent integrated water quality management, monitoring of AMR, and public health interventions to minimize infection risks and the spread of resistance in the community.

### Physico-chemical parameters of water quality

In this study, the pH of water samples ranged from 5.2 to 6.8; hence, water in Nekemte town was slightly acidic. Analyses of water availability throughout Ethiopia showed that the pH was within recommended limits. Tap and bottled waters in Addis Ababa also showed pH within acceptable limits. Comparatively, these studies indicate that water sources in Nekemte town are slightly more acidic compared to other locations within the country, presumably as a consequence of local geology or anthropogenic influences such as runoff, which contains organic acids (Table 4). The pH values are slightly lower than the WHO drinking water range of 6.5–8.5, though very close; similarly, the Ethiopian standard, ES 261:2001, classifies a permissible range near both ends. Long-term consumption of acidic water may enhance corrosion in distribution networks and make metals more soluble; hence, safety will be compromised. This result, therefore, pinpoints the need for continued monitoring and, where necessary, adjustment in water treatment practices to maintain safe drinking water quality within the area.

Water temperatures showed only a modest variation, ranging from 22 °C for the unprotected spring to 20 °C for tap water. Indeed, no obvious differences were apparent in the temperatures of water from the different sites sampled (Table 4). These temperatures agree with earlier studies conducted in Gondar [20.0 °C; 13] and Nekemte [20.8 °C; 7] and are below the maximum limits set by WHO^15^. The low variability may be related to the tropical climate, characterized by prevailing high ambient temperatures and considerable rainfall that could influence the thermal characteristics of the water and presumably also affect microbial activities within these sources of water.

Generally, electrical conductivity, being an indicator of total dissolved salts, was below the limits as indicated by WHO in all sampled water sources, reflecting low ionization with less mineral content. Comparatively, fairly higher values of electrical conductivities have been reported in other Ethiopian cities^16,17^. probably due to geological setting, leaching from fertilizers and pesticides, or characteristics of the soil. In this context, TDS ranged between 40 and 120 mg/L in the sampled water sources, hence still below the acceptable limit according to WHO^18^. The value of TDS recorded in Chelaleki unprotected spring was the lowest, while the highest (120 mg/L) was recorded in a protected spring, reflecting variable contents of minerals subject to local environmental conditions (Table 4).

TSS analyses gave very low values from all water sources, showing that TSS is not a major contributor to the pollution of water in the city of Nekemte. Although unprotected springs water with TSS values over 100 mg/L but below 220 mg/L is categorized as medium-polluted wastewater, most samples in this study fell far below this threshold and, hence, indicated minimal impact from surface runoff or domestic sewage (Table 4). Another interesting relationship was seen in the downstream increases of total solids and decreases in both EC and TDS, suggesting particulate matter settles along the course of the unprotect spring and thus could affect measurements of dissolved minerals. However, TSS does not seem to be the major concern regarding water quality in the sources sampled, and microbial contamination or chemical pollutants may play a more important role. This means that the general physicochemical parameters of water sources in Nekemte town are within the acceptable limits, except for minor acidity and variation in mineral content. Though these conditions are not immediately hazardous, they are related to the processes of corrosion, metal solubility, and microbial survival, and so indicate a need for continued monitoring and possible treatment.

**Table 4:** Physiochemical analysis (pH, electrically conductive, total dissolved substances, total solid and temperatures) of different water sources In East Wollega Zone, Nekemte town 2020.

Sites	Sources	pH	EC	TDS	TSS	Temperature
Cheleliki	Tap water	6.37 ± 0.05^a^	77.33 ± 0.47^d^	50.03 ± 0.05^d^	77.33 ± 0.47^bc^	20.17 ± 0.85^a^
	Unprotected spring	6.07 ± 0.04^a^	44.43 ± 0.09^ef^	28.00 ± 0.82^fg^	52.67 ± 0.94^de^	20.50 ± 0.41^a^
	Protected spring	6.60 ± 0.28^a^	173.23 ± 0.21^ab^	113.17 ± 0.24^b^	54.33 ± 1.25^de^	21.00 ± 0.82^a^
Darge	Tap water	6.83 ± 0.12^a^	64.73 ± 0.52^de^	42.33 ± 0.47^de^	41.00 ± 0.82^ef^	19.73 ± 0.52^ab^
	Unprotected spring	5.90 ± 0.08^ab^	63.00 ± 0.82^de^	40.63 ± 0.45^de^	42.00 ± 0.82^ef^	20.83 ± 1.03^a^
	Protected spring	6.40 ± 0.14^a^	185.00 ± 0.82^a^	120.50 ± 0.41^a^	46.19 ± 0.60^ed^	20.33 ± 0.24^a^
Jato	Tap water	6.67 ± 0.24^a^	60.27 ± 0.38^de^	40.30 ± 0.50^de^	35.93 ± 0.74^e^	20.33 ± 0.24^a^
	Unprotected spring	6.17 ± 0.24^a^	58.33 ± 1.25^df^	36.00 ± 0.82^ef^	37.63 ± 0.52^e^	21.67 ± 1.25^a^
	Protected spring	5.13 ± 0.09^ab^	150.17 ± 0.24^c^	97.83 ± 0.24^c^	98.50 ± 1.08^a^	21.33 ± 0.47^a^

Note: Means with the same superscript letters within a column are not significantly different (*p* > 0.05), based on one-way ANOVA followed by Tukey’s HSD post-hoc test.

## Conclusion

The results from the bacteriological tests conducted on the water sources in the town of Nekemte indicated that microbial contamination was common in the town’s water sources, especially in water from the unprotected springs. For instance, the total coliforms (TC), fecal coliforms (FC), Enterobacterium bacteria (EBC), and heterotrophic plate counts (HPC) of the town’s water sources were greater than the permissible levels set by the World Health Organization and the United States Environmental Protection Agency in almost all the town’s natural water sources. For instance, the HPC of the town’s water sources, ranging from the Jato Protected Spring, was between 34 CFU/ 100 mL and 282 CFU/100 mL in the town’s Hawato Protected Spring, whereas the FC was up to 2.89 log CFU/100 mL. The bacterial content in the town’s drinking water was low, whereas the water from the bottles was free from bacterial contamination. Furthermore, the town’s water sources were also found to contain pathogenic bacteria, e.g., *E. coli*,* Citrobacter*,* Klebsiella*,* Staphylococcus aureus*, and *enterococci*, in the town’s water sources.

In addition, the presence of Gram-positive cocci, which include *Staphylococcus aureus* and *enterococc*i species such as faecalis, indicates the risk associated with waterborne pathogens in water sources. The antibiotic susceptibility test revealed that the isolates were highly resistant to Oxacillin, Amoxicillin, Ampicillin, and Erythromycin, while Ciprofloxacin, Chloramphenicol, and Gentamicin were found to be effective in inhibiting the bacteria, indicating the presence of multidrug-resistant bacteria. Misuse of antibiotics could contribute to the development of antibiotic resistance.

Physicochemical water quality parameters were within acceptable limits, slightly acidic, ranging from 5.2 to 6.8, EC ranging from 44 to 185 microsiemens/cm, TDS ranging from 28 to 120 mg/L, and TSS, which was low, indicating minimum chemical pollution but the possibility of corrosion problems and solubility of metals.

### Recommendations

- Strengthen water treatment of tap water, and protect natural springs strictly.
- Regularly monitor bacteriological and physicochemical water quality, including surveillance of antimicrobial resistance.
- Raise public awareness about the safe handling of water, including discouraging the consumption of untreated water.
- Strengthen water quality standards, including antimicrobial stewardship programs, to restrict the environmental spread of resistant bacteria.
- Conduct further studies to identify the sources of water contamination, including effective intervention strategies.

## Data Availability

The datasets used and/or analyzed during the current study available from the corresponding author on reasonable request.
